# A method using electroporation for the protein delivery of Cre recombinase into cultured *Arabidopsis* cells with an intact cell wall

**DOI:** 10.1038/s41598-018-38119-9

**Published:** 2019-02-15

**Authors:** Yuichi Furuhata, Ayako Sakai, Tomi Murakami, Mone Morikawa, Chikashi Nakamura, Takeshi Yoshizumi, Ushio Fujikura, Keiji Nishida, Yoshio Kato

**Affiliations:** 1 Biomedical Research Institute, National Institute of Advanced Industrial Science and Technology (AIST), 1-1-1 Higashi, Tsukuba, 305-8566 Japan; 2 Department of Biotechnology and Life Science, Tokyo University Agriculture and Technology, 2-24-16 Naka-cho, Koganei, Tokyo 184-8588 Japan; 3 Biomacromolecules Research Team, RIKEN Center for Sustainable Resource Science, 2-1 Hirosawa, Wako-shi, Saitama 351-0198 Japan; 4 Graduate School of Science, Technology and Innovation, Kobe University, 1-1 Rokkodai-cho, Nada-ku, Kobe, Hyogo 657-8501 Japan

## Abstract

Genome engineering in plants is highly dependent on the availability of effective molecular techniques. Despite vast quantities of research, genome engineering in plants is still limited in terms of gene delivery, which requires the use of infectious bacteria or harsh conditions owing to the difficulty delivering biomaterial into plant cells through the cell wall. Here, we describe a method that uses electroporation-mediated protein delivery into cultured *Arabidopsis thaliana* cells possessing an intact cell wall, and demonstrate Cre-mediated site-specific recombination. By optimizing conditions for the electric pulse, protein concentration, and electroporation buffer, we were able to achieve efficient and less-toxic protein delivery into *Arabidopsis thaliana* cells with 83% efficiency despite the cell wall. To the best of our knowledge, this is the first report demonstrating the electroporation-mediated protein delivery of Cre recombinase to achieve nucleic acid-free genome engineering in plant cells possessing an intact cell wall.

## Introduction

Genome engineering is a powerful molecular tool that has been extensively used in numerous areas of the life sciences. One of the most popular genome engineering tools is Cre recombinase, which catalyzes recombination between two of its consensus DNA sequences, named *loxP*^1–3^. The Cre/loxP system has served as a versatile platform for lineage/cell type-specific gene knockout, selectable marker removal, and chromosomal rearrangement^4–8^, and has been widely used in a variety of organisms (e.g., drosophila, mice, and humans)^9–11^. Although the Cre/loxP system has also been used in plant cells^12–17^, its application has been limited compared to that in animal cells. One major obstacle is the difficulty of the temporal delivery of biomaterial into plant cells.

The genome engineering of cultured cells is highly important for plant research in assessing the function of target genes. In comparison to analyses using whole plants, research using cultured cells enables us to analyze gene functions more quickly and reduces variation at the level of the individual. However, the genome engineering of cultured plant cells is limited owing to the difficulty of delivering biomaterials across the cell wall, which provides plant cells with protection against mechanical stress and the entry of exogenous substances^18^. Therefore, the delivery of biomaterials into plant cells has been widely considered to be more difficult than delivery into cells derived from other organisms (i.e., lacking a cell wall). One way to work around this limitation is to use protoplasts whose cell walls are enzymatically removed by cellulase and/or pectinase. Several studies have shown that exogenous biomaterials such as DNA, RNA, and protein can be successfully delivered into protoplasts generated from various plant organisms such as tobacco, *Arabidopsis*, and rice^19–22^. However, the regeneration of whole plants from protoplasts has been shown to be either impossible or inefficient in many plant species owing to the sensitivity to biochemical or mechanical stress^23,24^.

In order to introduce genetic materials into plant cells through the cell wall, the delivery of DNA using a particle gun or *Agrobacterium* (*Rhizobium*)-mediated techniques are widely used in plant research^25,26^. Although these methods enable the efficient expression of genome engineering proteins, including Cre recombinase, exogenous DNA fragments that are introduced using a particle gun or *Agrobacterium* are often or invariably incorporated into the genomic DNA, and may induce unexpected effects that interfere with subsequent analyses. In addition, the success of *Agrobacterium*-mediated delivery is strongly dependent on the combination of the bacterial strain and the plant species or variety. To overcome these obstacles, a method for the delivery of proteins directly into plant cells possessing a cell wall is in high demand. Several groups have attempted the delivery of proteins into *Chlamydomonas* and tobacco cells through the cell wall using electroporation^27–29^. However, the delivery efficiency was either not quantified or was very low, and little is known about whether the delivered proteins are functionally active in the cell nucleus and cytoplasm. Although Cao *et al*. demonstrated the delivery of a modified Cre protein into rice calli after protoplasting or plasmolyzing and achieved regeneration of rice plants with Cre-excised target sequence^15^, the delivery efficiency was insufficient or harsh for the biological analysis of cultured plant cells.

Here, we demonstrate an efficient electroporation technique for the delivery of proteins into cultured plant cells that possess a cell wall. Using *Arabidopsis thaliana* as a model, we constructed a reporter cell-line that responds to Cre recombinase and then expresses the gene for ß-glucuronidase (GUS), which enables us to quantify the delivery efficiency. By optimizing the conditions for the electric pulse, protein concentration, and electroporation buffer, we successfully achieved efficient and less-toxic protein delivery in 83% of *Arabidopsis thaliana* cells, despite the cell wall. To the best of our knowledge, this is the first report to demonstrate the electroporation-mediated protein delivery of Cre recombinase to achieve nucleic acid-free genome engineering in plant cells possessing a cell wall.

## Methods

### Preparation of Cre protein

Cre proteins were expressed using *Escherichia coli* (*E. coli*) strain BL21(DE3) carrying the plasmid pET-HNCre(A207T)^30^, which encodes a hexa-histidine tag at the N-terminus followed by an SV40 nuclear localization signal and Cre recombinase (see Supplementary Figure 1). Our Cre gene carries the A207T mutation, whose recombination activity is comparable to the wildtype (Supplementary Figure 2). *E. coli* was cultured at 37 °C for 3 h with shaking. Protein expression was then induced using 0.1 mM isopropyl β-D-1-thiogalactopyranoside (IPTG). After 2.5 h, cells were lysed with lysis buffer (50 mM Tris-HCl, 500 mM NaCl, 10% glycerol, 10 mM imidazole, 1 mM benzylsulfonyl fluoride, 1 mM dithiothreitol, pH 8.0). HNCre proteins were purified using an Ni-NTA column with washing buffer (50 mM Tris-HCl, 500 mM NaCl, 10% glycerol, 20 mM imidazole, pH 8.0) and eluted with elution buffer (50 mM Tris-HCl, 500 mM NaCl, 10% glycerol, 500 mM imidazole, pH 8.0). HNCre proteins were further purified using a gel filtration column (HiPrep 16/60 Sephacryl S-200 HR; GE healthcare, Chicago, IL, United States) using Buffer A (20 mM HEPES, 500 mM NaCl, 10% glycerol, 1 mM dithiothreitol, pH 7.4). Proteins were flash-frozen in liquid N_2_ and stored at −80 °C. Frozen proteins were thawed and dialyzed with HBS (20 mM HEPES, 150 mM NaCl, 5 mM KCl, 25 mM glucose) immediately before use.

### Plasmid construction

To construct pCAMBIA-N-xGxGUS, the NOS promoter was amplified with primers (ACGGCCAGTGCCAAGCTTGATCATGAGCGGAGAATTAAG and TCTGCGAAAGCTCGACCTAGGAAACGATCCAGATCCGGTGCA) from pRI201 (TaKaRa Bio. Inc., Shiga, Japan). The resulting fragment was cloned using the In-Fusion HD Cloning Kit (Takara Bio) into pCAMBIA 1305.2 (Marker Gene Technologies Inc., Eugene, OR, United States), which had been partially digested with HindIII and XhoI. The GFP fragment (mEmerald) was sandwiched between two *loxP* sites, and was subsequently amplified using primers (GGACTCTTGACCATGTAATAACTTCGTATAGCATACATTATACGAAGTTATGTTAACTACATCACAATCACACAAAAC and TTAGTAGTAGCCATGGTCTAGATAACTTCGTATAATGTATGCTATACGAAGTTATGGGCCCCTTATCTTTAATCATATTCCA) from pcDNAFRTxE2CxmEm^30^. The resulting fragment was cloned into the NcoI site using the In-Fusion HD Cloning Kit. To construct pCAMBIA-B-HNCre(A207T), the *Bsd*^*R*^ gene was amplified using primers (ATCTATCTCTCTCGACCTAGGTTGATAGATATGGGCCAGGCCAAGCCTTTGT and TATGGAGAAACTCGATTTAAATTAGCCCTCCCACACATAACCAGA) that were originally from pTracer-EF/Bsd (Thermo Fisher Scientific, Waltham, MA, United States). The resulting fragment was cloned by In-Fusion into pCAMBIA 1305.2, which had been digested with XhoI. The Cre fragment was then amplified using primers (GGACTCTTGACCATGGGCCACCATCACCAC and ATTCGAGCTGGTCACCCGTCGACGTTAATCGCCATCTTCCAGCAG) from pFT-HNCre(A207T), and the resulting fragment was cloned by In-Fusion between the BstEII and NcoI sites. Please also refer to Supplementary Figure 1.

### Cell materials and culture

The *Arabidopsis thaliana* T87 cell line was obtained from RIKEN Bio Resource Center (Ibaraki, Japan) and cultured in a liquid NT1 culture medium (30 g/L sucrose, 0.1 mM KH_2_PO_4_, 1 × Murashige Skoog Salt Mixture and Vitamins, 2 µM 2,4-dichlorophenoxyacetic acid, pH 5.8 adjusted with KOH) at 22 °C while shaking under light, unless otherwise specified. Cells were maintained by 15-fold weekly dilutions. *Agrobacterium tumefaciens* (*Rhizobium radiobacter*) strain GV3101 was cultured in LB medium (Merck, Darmstadt, Germany) supplemented with 50 µg/mL rifampicin and 30 µg/mL gentamicin at 28 °C with shaking. To prepare the transformants, GV3101 cells were electroporated with the indicated binary plasmids and cultured in LB medium supplemented with 50 µg/mL rifampicin, 30 µg/mL gentamicin, and 50 µg/mL kanamycin. Prior to infection, GV3101 cells carrying the indicated binary plasmids were inoculated into LB medium supplemented with 50 µg/mL rifampicin, 30 µg/mL gentamicin, and 50 µg/mL kanamycin. After an overnight culture, cells were washed thrice with liquid B5 medium (30 g/L sucrose, 0.5 g/L MES, 1x Gamborg’s B5 Salt Mixture and Vitamins, and 1 µM 1-naphthaleneacetic acid) and resuspended to a final OD_600_ of 0.6.

### *Agrobacterium*-mediated transformation of T87 cells

Cells were transferred into liquid B5 medium and cultured for 2 days. The cells containing 30 µL of packed cell volume (PCV) were then infected with 0.8 µL of GV3101 inoculate (OD_600_ of 0.6) carrying the indicated binary plasmids with 200 µM acetosyringone. Two days after infection, the cells were washed thrice using liquid B5 medium supplemented with 200 µg/mL cefotaxime to eliminate GV3101, and further cultured in liquid B5 medium supplemented with 200 µg/mL cefotaxime for 3 days. For the construction of a reporter T87 cell line, the cells were additionally transferred to CIM agar (0.6% agar, 30 g/L sucrose, 0.5 g/L MES, 1x Gamborg’s B5 Salt Mixture and Vitamins, 1 µM 1-naphthaleneacetic acid, pH 5.7 adjusted with KOH) with 200 µg/mL cefotaxime and 20 µg/mL hygromycin and cultured for 2 weeks. A single clone, exhibiting bright green fluorescence, was picked and cultured in liquid NT1 medium containing 10 µg/mL hygromycin, and designated as the T87-xGxGUS cell line. T87-xGxGUS cells were maintained in the same way as T87 cells, using the liquid NT1 medium containing 10 µg/mL hygromycin.

### Electroporation

T87-xGxGUS cells were 15-fold diluted into fresh NT1 medium 1–5 days before electroporation. On the day of electroporation, cells were collected in a tube and washed once with the electroporation solution: Opti-MEM I (Thermo Fisher Scientific), PBS (phosphate buffered saline, Thermo Fisher Scientific), NT1, B5 medium, deproteinized Opti-MEM I, MEM (Minimal Essential Medium, Thermo Fisher Scientific). The deproteinization of Opti-MEM I was performed using 3 kDa filter (Sartorius). Cells with 20 µL of PCV were suspended in 200 µL of electroporation solution with the indicated concentration of Cre protein on ice, and transferred into a 4-mm-wide cuvette (Nepa Gene, Chiba, Japan). The standard electroporation program was 375 V/cm (150 V setting/0.4 cm width), 10 ms, 5 times, and a 50 ms interval for poring pulse, and 50 V/cm (20 V setting/0.4 cm width), 50 ms, 20 times, and a 50 ms interval for transfer pulse. Electroporation was performed using NEPA21 TypeII (Nepa Gene). Directly after electroporation, cells were washed once with NT1 medium and cultured in 2 mL of NT1 medium at 22 °C with shaking until the assay. To assess the effect of the size of cell aggregates on electroporation efficiency, cell aggregates were fractionated into small (<100 μm), medium (100–300 μm), and large (>300 μm) using 100 μm nylon mesh (Falcon, Corning, Tewksbury, MA, United States), and a 300 μm polyethylene terephthalate mesh (pluriSelect Life Science, Leipzig, Germany) before electroporation.

### Preparation of protoplasts

T87-xGxGUS cells were washed with 400 mM mannitol. Then the supernatant was discarded and replaced with the enzyme solution. The enzyme solution consisted of 20 mM MES (pH 5.7), 400 mM mannitol, 20 mM KCl, 10 mM CaCl_2_, 1.5% (w/v) Onozuka RS (Serva Electrophoresis, Heidelberg, Germany), and 0.25% (w/v) Macerozyme R-10 (Yakult Pharmaceutical Industry). The suspension of T87-xGxGUS cells in the enzyme solution was incubated at 22 °C with shaking in the dark. After 4 h of incubation, CaCl_2_ (final concentration of 77 mM) was added.

### Evans blue staining

Cytotoxicity was analyzed by staining with Evans blue 1 h after electroporation. Cells were washed once with water and incubated with 0.05% (w/v) Evans Blue (FUJIFILM Wako Pure Chemical, Osaka, Japan) staining solution for 15 min at RT. Cells were then washed once with water. Images were obtained using a phase contrast microscope (Nikon, Tokyo, Japan). For quantification, the supernatant was discarded, and Evans blue stain was extracted using 50% methanol 1% SDS. Absorbance at 595 nm was measured spectrophotometrically using a NanoDrop ONE (Thermo Fisher Scientific).

### GUS staining

To visualize GUS expression, cells were incubated with 0.5 mg/mL of X-Glucuronide (X-Gluc, Carbosynth, Berkshire, United Kingdom) dissolved in staining buffer (20% (w/v) methanol, 50 mM NaH_2_PO_4_, pH 7.0) for 30 min at 37 °C. For the staining of protoplasts, mannitol (final concentration of 400 mM) was added to the GUS staining solution. Cells were then transferred into 70% (w/v) ethanol and images were obtained using a phase contrast microscope (Nikon).

### Fluorescence quantification of GUS activity

Two days after electroporation, cells were washed once with NT1 medium (pH 7.0; adjusted with KOH). Cells with 5 µL of PCV were then suspended in 100 µL of NT1 medium (pH 7.0) containing 10 µM 6-chloro-4-methylumbelliferyl β-D-glucuronide (CMUG; Glycosynth, Cheshire, United Kingdom) and transferred to a black 96-well plate (Thermo Fisher Scientific). Fluorescence was measured using a Wallac 1420 ARVOsx microplate reader (PerkinElmer, Waltham, MA, United States). The excitation wavelength was set at 355 nm and the emission wavelength at 460 nm. Chlorophyll was quantified to normalize GUS activity. For the chlorophyll extraction, cells with 5 µL of PCV were immersed in N,N-dimethylformamide for over 6 h at 4 °C in the dark. Absorbance at 646 and 664 nm was measured using a NanoDrop ONE. The amount of chlorophyll was then calculated according to a previous study^31^. GUS activity is obtained as the increase in fluorescence intensity per minute per microgram of chlorophyll a and b.

### Genomic PCR of Cre-responsive reporter cassette

Two days after electroporation, cells were incubated with 10 µL of 250 mM NaOH and 0.1% Tween20 at 98 °C for 10 min to extract genomic DNA. Cells were then added with 5 µL of 1 M Tris-HCl [pH6.5] and subsequently centrifuged. Using the extracted genomic DNA, the integrated sequence of Cre-responsive reporter cassette in T87 genome was amplified via PCR using MightyAmp DNA Polymerase Ver.3 (Takara Bio.) (98 °C for 2 min; 30 cycles of 98 °C for 10 s, 60 °C for 15 s, and 68 °C for 2 min) with the primers TCCTTCGCAAGACCCTTCCTC and GGATGGCAAGAGCCAAATGCTTAG. The PCR products were analyzed by agarose gel electrophoresis. Gels were stained with GelGreen (Biotium, Hayward, CA, United States) and imaged using a ChemiDoc XRS + system (Bio-Rad, Hercules, CA, United States). Quantification was based on relative band intensities. The following equation was used to calculate the percent recombination by Cre protein: 100 × (a/[a + b]), where a or b is the intensity of the PCR product with or without recombination, respectively.

### Quantification and Statistical Analyses

All measurements are presented as mean ± standard error (SE). Sample sizes are indicated in the figure legends.

## Results

### Establishment of reporter T87 cell line

Protein delivery into cells is a promising approach for biotechnology, and thus has been extensively developed in mammalian cells. However, this technique has not been widely used in plants thus far, as the penetration of the cell wall and validating the successful introduction of protein into cells has proven to be problematic. Because of the chloroplast—a common feature of the vast majority of plants—fluorescently-labeled proteins are difficult to observe owing to autofluorescence from chlorophyll. Furthermore, the visualization of fluorescently-labelled biomaterials in fixed tissues or cells results in a substantial degree of artifacts owing to fixation^32^. Recently, Cedeño *et al*. electroporated a stress-related protein ERD14 into the cell cytoplasm of tobacco BY-2 cells^29^, although the microscopic observation of a fluorescent probe does not unequivocally prove that intracellular delivery was successful, as the proteins fused with fluorescent proteins do not often reflect their original localization^33^. In addition, if the protein of interest can exert its function outside of the cell nucleus or cytoplasm, such as the endosome or intercellular gap, the protein may appear but not actually localize to the cell nucleus or cytoplasm^34^. Thus, one must pay special attention to proving the intracellular delivery of biomaterials.

**Figure 1:**
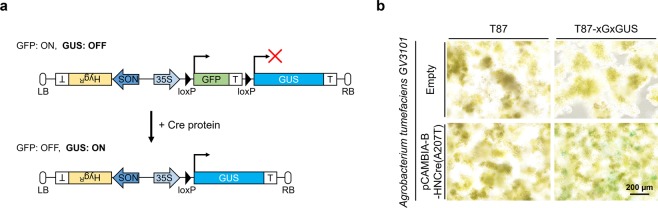
Design of reporter T87 cell line, T87-xGxGUS. (**a**) Schematic of Cre reporter design before and after Cre-mediated recombination. T, Hyg^R^, GFP, GUS, LB, and RB indicate terminator polyadenylation signal, hygromycin-resistant gene, green fluorescent protein, ß-glucuronidase, left border, and right border, respectively. (**b**) Cre-dependent reporter activity in T87-xGxGUS cells. T87 and T87-xGxGUS cells were infected with *Agrobacterium tumefaciens* GV3101 carrying an empty vector or pCAMBIA-B-HNCre(A207T). GUS staining was performed at 5 days after infection without antibiotic selection. Scale bar represents 200 μm.

To verify the intracellular delivery of proteins in *Arabidopsis thaliana* cells, we adopted a Cre protein system, which is only active when introduced into the cell nucleus. To evaluate the activity of the intracellular delivery of Cre proteins, we first established a reporter cell line (T87-xGxGUS) by stably integrating part of pCAMBIA-N-xGxGUS—a binary plasmid encoding green fluorescent protein (GFP), followed by a transcription termination signal and a sequence encoding GUS—into the genome of the *Arabidopsis thaliana* T87 cell line using *Agrobacterium* (Fig. 1a). Once Cre recombinase is introduced into the cell nucleus and the intervening sequence between two directly oriented *loxP* sites is excised, T87-xGxGUS cells—originally expressing GFP—come to express GUS, which can be stained with blue dye using X-Gluc or produces a fluorescent product from CMUG (Fig. 1a). To test whether the reporter cells work, cells were infected with *Agrobacterium* carrying pCAMBIA-B-HNCre(A207T), a binary plasmid encoding Cre recombinase under the 35 S promoter. Three days after infection, some of the cells exhibited GUS expression upon Cre recombination (Fig. 1b). This result demonstrates that T87-xGxGUS cells enable us to validate the intracellular delivery of the Cre protein.

### Electroporation of Cre protein into T87 cells with an intact cell wall

**Figure 2:**
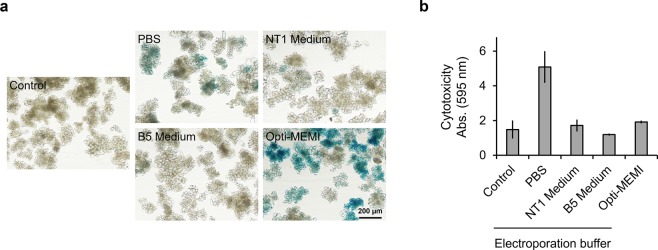
Electroporation-mediated Cre protein delivery into T87 cells. (**a**) GUS staining of T87-xGxGUS cells electroporated using the indicated buffer. Cells were electroporated in the indicated buffer containing 1 μM Cre protein and 5 poring pulses of 375 V/cm for 10 ms. GUS staining was performed at 2 days after electroporation. Control indicates untreated T87-xGxGUS cells. Scale bar represents 200 μm. (**b**) Cytotoxicity of electroporation. Evans blue staining was performed at 1 hour after electroporation. Evans blue was extracted using 50% methanol/1% SDS and the absorbance at 595 nm was measured. Values shown are the mean ± SE of n = 3.

Next, we attempted to deliver Cre protein into T87 cells with an intact cell wall. NEPA21 TypeII was used for the electroporation. NEPA21 TypeII generates a square wave pulse, which yields higher viabilities of electroporated cells in comparison to an exponential decay wave pulse. GUS activity was assessed 2 days after electroporation. Additionally, cell viability was tested by Evans blue staining 1 hour after electroporation. We tested several buffers (e.g., NT1 medium (modified MS), B5 medium, PBS, and Opti-MEM I) for electroporation. The electric resistivity of the four electroporation buffers—NT1 medium, B5 medium, PBS, and Opti-MEM I—were 1.86, 2.92, 0.850, and 0.903 Ω⋅m, respectively. As shown in Fig. 2a, there were few GUS-expressing cells when electroporation was performed using NT1 medium or B5 medium. Although some cells expressed GUS when electroporated using PBS, high cytotoxicity was observed in this condition (Fig. 2b). Surprisingly, when Opti-MEM I was used as an electroporation buffer, most cells expressed GUS with less cytotoxicity (Fig. 2a and b). As demonstrated by these experiments, we successfully accomplished electroporation-mediated protein delivery into T87 cells with an intact cell wall using Opti-MEM I. To investigate which components of Opti-MEM I promote the electroporation efficiency, we tested two additional buffers [deproteinized Opti-MEM I and Minimal Essential Medium (MEM)], since Opti-MEM I is a modified MEM and contains two proteins (insulin and transferrin), hypoxanthine, thymidine, and trace elements. The cells electroporated using Opti-MEM I exhibited equivalent effect compared to those using deproteinized Opti-MEM I and MEM in terms of both GUS activity and cytotoxicity (Supplementary Figure 3). These buffers contain not only inorganic salts but also organic components such as amino acids and vitamins, mimicking intracellular conditions (Supplementary Table). The presence of these components could reduce cytotoxicity. In addition, we examined the effectiveness of another electroporation system called Nucleofector. As shown in Supplementary Figure 4, Cre proteins were successfully delivered using Nucleofector when Opti-MEM I was used as an electroporation buffer.

### Effect of poring pulse conditions on electroporation efficiency

**Figure 3:**
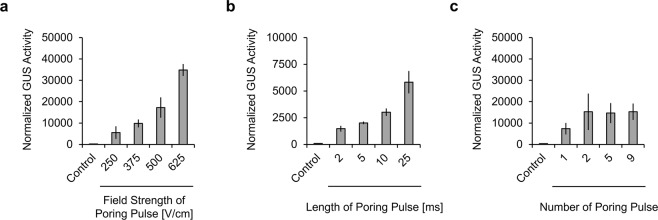
Effect of poring pulse conditions on electroporation efficiency. (**a**–**c**) Normalized GUS activity as determined by the fluorescent measurement of catalyzed CMUG in electroporated T87-xGxGUS cells using different poring pulse conditions. (**a**) Field strength (250, 375, 500, or 625 V/cm), (**b**) duration (2, 5, 10, or 25 ms), and (**c**) number (1, 2, 5, or 9) of poring pulses were changed from the standard poring pulse conditions (375 V/cm, 10 ms, 5 times). In all cases, 1 μM Cre protein dissolved in Opti-MEMI was used for electroporation. Control indicates untreated T87-xGxGUS cells. Two days after electroporation, GUS activity was measured and normalized to the amount of chlorophyll. Values shown are the mean ± SE of n = 3.

As the electroporation efficiency is largely affected by the electric condition of the poring pulse, we decided to assess the effect of poring pulse conditions using the NEPA21 TypeII electroporation system. The three main parameters of poring pulse are: field strength, duration, and the number of pulses. The standard poring pulse conditions, used in Fig. 2a, was 5 poring pulses of 375 V/cm for 10 ms. To evaluate the effect of variations in these parameters, we set the various conditions as follows: field strength (250, 375, 500, or 625 V/cm), pulse duration (2, 5, 10, or 25 ms), and the number of poring pulses (1, 2, 5, or 9). To quantify the electroporation efficiency, we measured the enzymatic activity of GUS using CMUG, and normalized it to the amount of chlorophyll. As shown in Fig. 3a, the normalized GUS activity of T87-xGxGUS cells increased concomitantly with the field strength of the poring pulse, although cytotoxicity also increased in response to increases in field strength (Supplementary Figure 5a). While the normalized GUS activity increased concomitantly with the duration of poring pulse (Fig. 3b), cytotoxicity was nearly unchanged within the range of 2–10 ms (Supplementary Figure 5b). The number of poring pulses did not affect normalized GUS activity at more than 2 pulses (Fig. 3c), while cytotoxicity did not increase at greater than 1 pulse (Supplementary Figure 5c). These results indicate that the field strength and duration of poring pulse are important factors for protein electroporation into *Arabidopsis thaliana* cells, and that more protein can be electroporated by increasing the field strength or/and duration of poring pulse.

### Effect of the size of cell aggregates on electroporation efficiency

**Figure 4:**
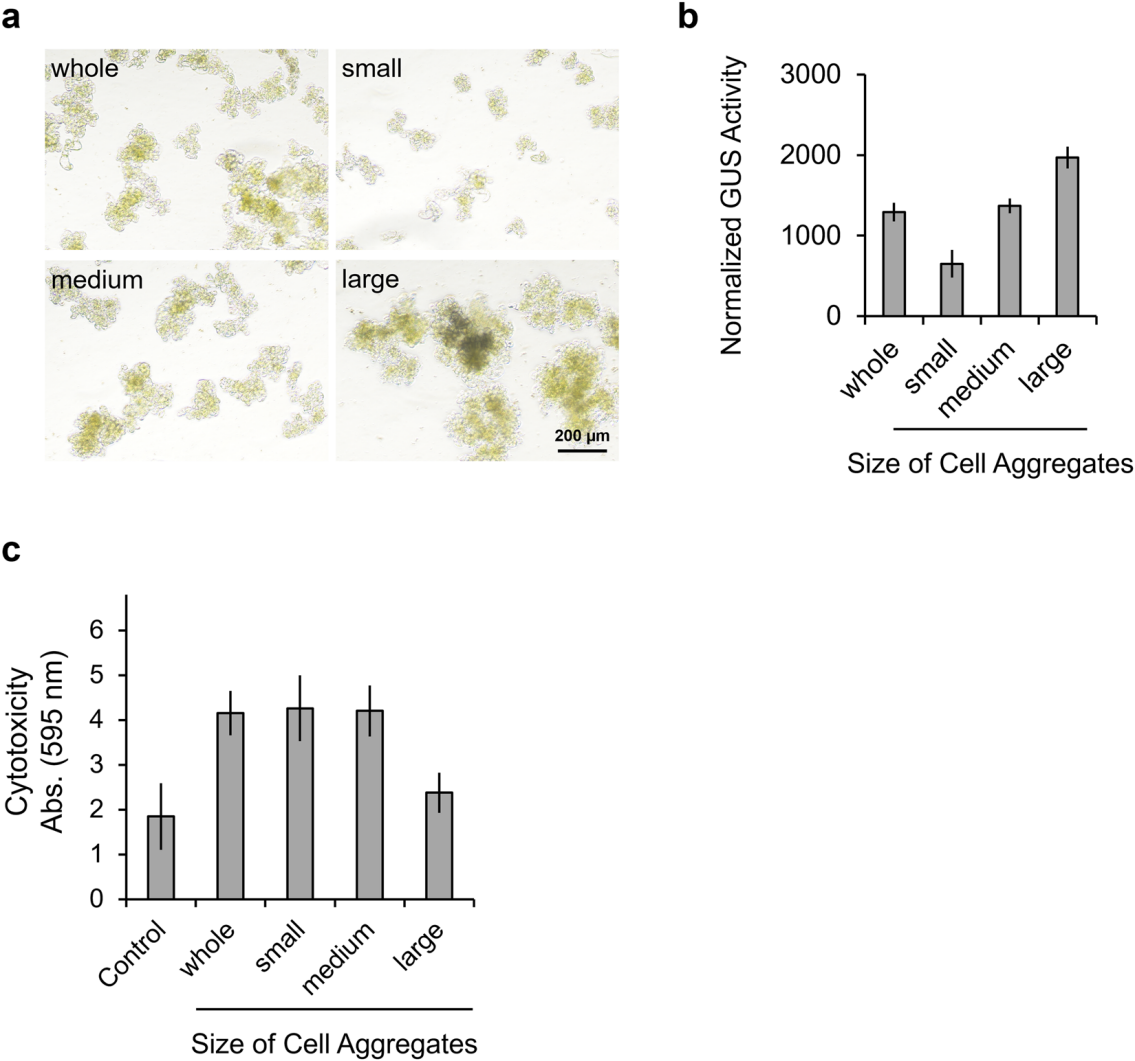
Effect of cell aggregation on electroporation efficiency. (**a**) Images of fractionated T87-xGxGUS cells. T87-xGxGUS cell aggregates were fractionated into small (<100 μm), medium (100–300 μm), and large (>300 μm) according to their size. Whole indicates unfractionated T87-xGxGUS cell aggregates. Scale bar represents 200 μm. (**b**) Normalized GUS activity as determined by the level of fluorescence of catalyzed CMUG in electroporated cell aggregates of different sizes. Cells were electroporated with 1 μM Cre protein in Opti-MEMI using 5 poring pulses of 375 V/cm for 10 ms. Two days after electroporation, GUS activity was measured and normalized to the amount of chlorophyll. Values shown are the mean ± SE of n = 3. (**c**) Cytotoxicity of electroporation. Evans blue staining was performed at 1 hour after electroporation. Evans blue was extracted using 50% methanol/1% SDS and the absorbance at 595 nm was measured. Control indicates untreated T87-xGxGUS cells. Values shown are the mean ± SE of n = 3.

In general, bacterial or mammalian cells are well-suspended by pipetting or by trypsinization to disperse the cells prior to electroporation, as surface area increases when cells are well-dispersed. Since cultured *Arabidopsis thaliana* cells form large aggregates as shown in Fig. 2a, aggregate size may affect delivery efficiency. To test this hypothesis, T87-xGxGUS cell aggregates were fractionated into small (<100 μm), medium (100–300 μm), and large (>300 μm) aggregates by passing through mesh of varying sizes prior to electroporation (Fig. 4a). Cells were then electroporated with 1 μM of Cre protein using the standard electroporation program and normalized GUS activity was measured. Surprisingly, the larger cell aggregates exhibited higher GUS activity, where the GUS activity of large aggregates was three times higher than that of small aggregates (Fig. 4b). The large aggregates are more likely to survive electroporation in comparison to small aggregates (Fig. 4c), consequently causing the ratio of GUS-expressing cells to increase in the large cell aggregates. This result suggests that *Arabidopsis thaliana* cells forming large aggregates, resembling callus or tissues, are suitable for protein electroporation.

### Effect of protein concentration on electroporation efficiency

**Figure 5:**
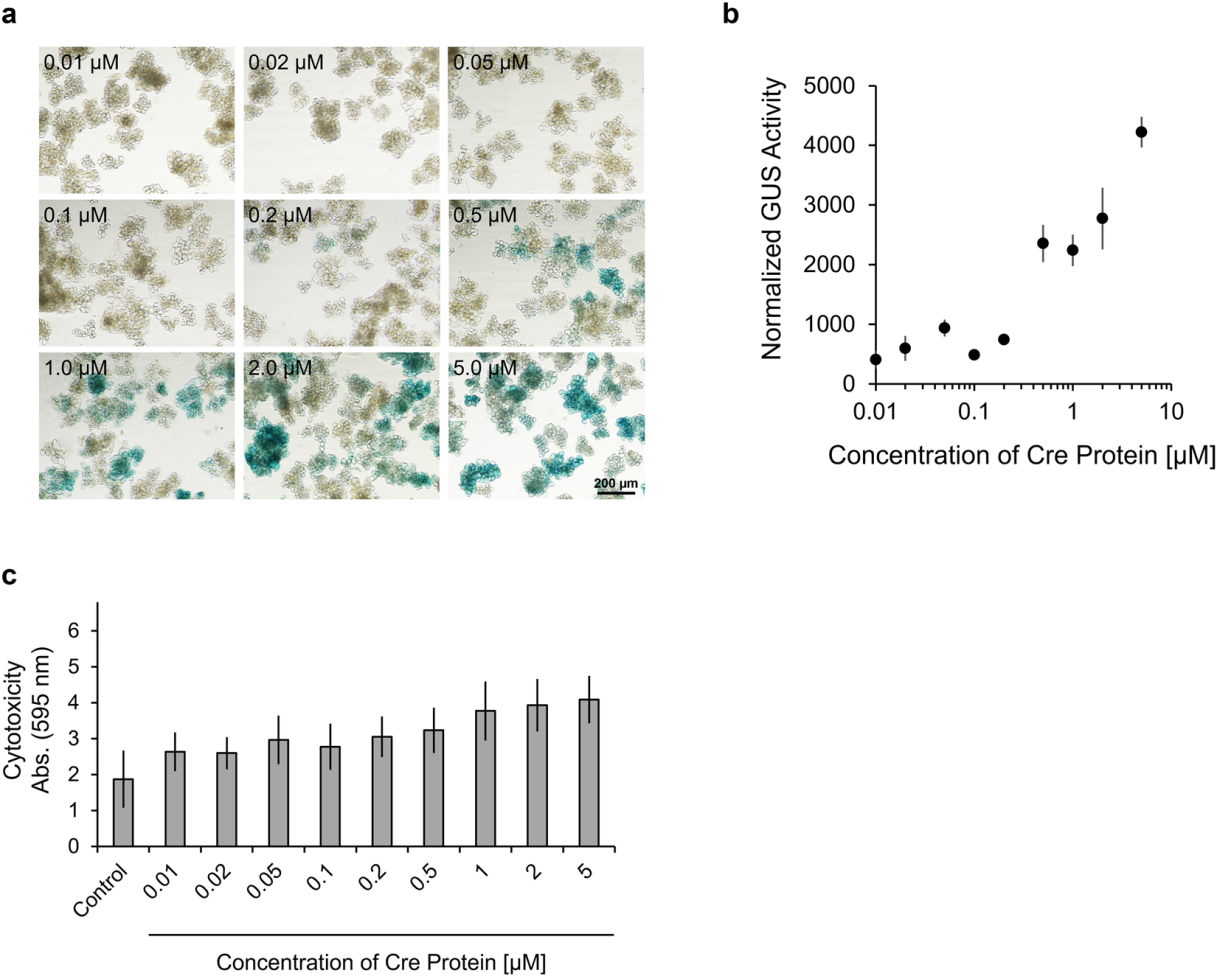
Effect of Cre protein concentration on electroporation efficiency. (**a**) GUS staining of T87-xGxGUS cells electroporated with different concentrations of Cre protein. Cells were electroporated with the indicated concentration of Cre protein in Opti-MEMI using 5 poring pulses of 375 V/cm for 10 ms. GUS staining was performed 2 days after electroporation. Control indicates untreated T87-xGxGUS cells. Scale bar represents 200 μm. (**b**) Normalized GUS activity as determined by the level of fluorescence of catalyzed CMUG in electroporated cells with different concentrations of Cre protein. Cells were electroporated with 0.01, 0.02, 0.05, 0.1, 0.2, 0.5, 1.0, 2.0, or 5.0 μM of Cre protein in Opti-MEMI using 5 poring pulses of 375 V/cm for 10 ms. Two days after electroporation, GUS activity was measured and normalized to the chlorophyll amount. Values shown are the mean ± SE of n = 3. (**c**) Cytotoxicity of electroporation. Evans blue staining was performed at 1 hour after electroporation. Evans blue was extracted using 50% methanol/1% SDS and the absorbance at 595 nm was measured. Control indicates untreated T87-xGxGUS cells. Values shown are the mean ± SE of n = 3.

The efficiency of the intracellular delivery of exogenous materials such as DNA, RNA, protein, and low-molecular compounds is highly dependent on the concentration of these materials. Therefore, we assessed the effect of Cre protein concentration on electroporation efficiency. Cells were electroporated with 0.01–5 μM of Cre protein using the standard electroporation program. GUS staining showed that the number of stained cells increased concomitantly with the concentration of Cre protein (Fig. 5a). The quantification of fluorescence also showed that GUS activity increased with increasing concentrations of Cre protein (Fig. 5b), while cytotoxicity was slightly increased concomitantly with the concentration of Cre protein (Fig. 5c). When electroporated with more than 0.2 μM of Cre protein, the cellular expression of GUS was substantial. These results reveal that protein concentration was an important factor in the efficacy of protein electroporation into *Arabidopsis thaliana* cells, where more than 0.2 μM of protein was required for efficient electroporation.

**Figure 6:**
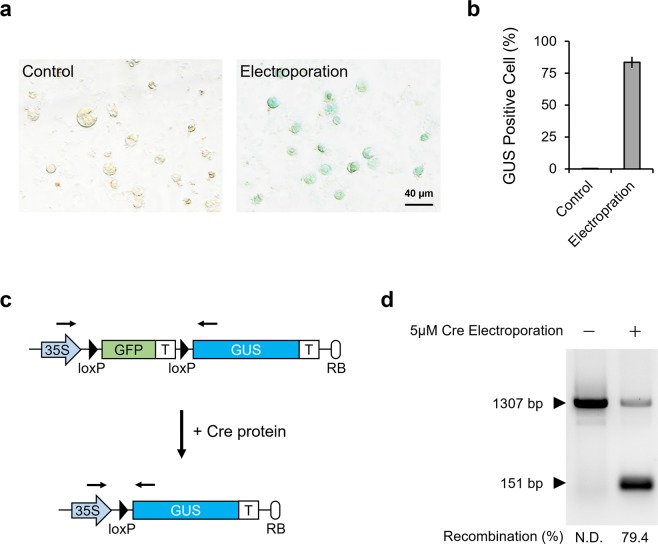
Quantification of protein delivery efficiency. (**a**) GUS staining of protoplasts constructed from electroporated T87-xGxGUS cells. Cells were electroporated with 5 µM of Cre protein, dissolved in Opti-MEMI, using 5 poring pulses of 375 V/cm for 10 ms. Protoplasts were constructed and stained 2 days after electroporation. Control indicates unelectroporated T87-xGxGUS cells. Scale bar represents 40 μm. (**b**) Quantification of the percentage of GUS positive cells from (**a**). Values shown are the mean ± SE of n = 3. In total, 254 and 288 cells were used for the quantification of control and electroporation samples, respectively. (**c**) Schematic of Cre reporter design before and after Cre-mediated recombination. Black arrows indicate primer binding sites. GFP and GUS indicate green fluorescent protein and ß-glucuronidase, respectively. (d) Agarose gel electrophoresis of genomic DNA PCR products from T87-xGxGUS cells. The 1307 bp and 151 bp fragments represent the reporter gene cassette before and after Cre-mediated recombination, respectively. A full-length gel image is presented in Supplementary Figure 6.

Furthermore, a detailed quantification of delivery efficiency was performed to clarify the population of cells in which protein had been successfully delivered. As almost all T87 cells form multicellular aggregates, cell dissociation is required to count the number of cells. To this end, we electroporated 5 μM of Cre protein to T87-xGxGUS cells (with intact cell walls), and then generated protoplasts using cellulase and pectinase to dissociate the cell aggregates. GUS staining revealed that 83% of cells expressed GUS (Fig. 6a and b). Next, we performed a genomic PCR analysis using primers that flanked the two *loxP* sites in the reporter cassette (Fig. 6c). As shown in Fig. 6d, electroporated cells exhibited a 79.4% recombination rate. This result is consistent with the fact that the frequency of GUS-expressing cells in all electroporated cells is approximately 83% (Fig. 6b). These results show that we successfully accomplished the efficient and less-toxic delivery of protein into *Arabidopsis thaliana* cells through the cell wall using electroporation, and most notably that the delivery efficiency was as high as 83%.

## Discussion

Effective techniques for the delivery of proteins of interest into cells are essential to biological research and bioengineering. The conventional method of introducing the DNA sequence for a given protein into a cell as a plasmid, and subsequently expressing the protein, has been widely accepted in the field of biology. However, vector-mediated protein expression often requires time-consuming steps, such as the optimization of codon and promoter sequences according to target cells. Furthermore, the delivered DNA frequently integrates into the host genome and can cause a variety of unintended side effects, such as unexpected gene disruption. As a result of the low level of stable integration by *Agrobacterium*-mediated gene delivery, transformants need to be selected using antibiotics. To address these problems, direct protein delivery—in which purified proteins are delivered into target cells without any DNA or RNA—has been attracting more and more attention. As proteins should be highly purified, this method substantially diminishes the risk of inserting exogenous DNA into the host genome, and eliminates the effort of codon and promoter optimization. Furthermore, in the case of genome engineering enzymes, as directly introduced proteins immediately modulate the target genome allow for temporary action in cells, the off-target effects of these enzymes can be reduced^35–37^. We have focused on methods of protein delivery and has introduced genome engineering enzymes, such as zinc-finger nuclease and Cre recombinase, to mammalian cells using direct delivery and nanoneedles^30,37,38^. Most notably, while genome engineering proteins are delivered for a relatively short period of time, the effects last permanently as the cell proliferates. Other research groups have also reported protein delivery methods for Cre recombinase, transcription activator-like effector nuclease (TALEN), and CRISPR-associated protein 9 (Cas9)^39,40^. However, to date, most of these techniques have been limited to animal cells.

It has been previously thought that the direct delivery of proteins was difficult in plant cells owing to the thick cell wall surrounding the cell membrane. Although a few studies have reported the successful delivery of proteins into plant protoplasts whose cell walls were enzymatically removed by cellulase and/or pectinase, these techniques require time-consuming steps and high-cost reagents owing to the number of enzymatic reactions. Moreover, whole plant regeneration from protoplasts is difficult for many plant organisms. Therefore, we decided to establish a method for the effective delivery of proteins into plant cells with the cell wall intact. Moreover, while it is well known that cell walls also exist in fungi and bacteria such as *E. coli*—which has been extensively used for plasmid cloning in the field of molecular biology—the constitutions of the cell walls of these organisms differ from those of plants. In the present study, we used Cre protein as the first demonstration of nuclear delivery in plants, where the biophysical nature of the protein—such as the size (40 kDa) and surface charges (isoelectric point of 9.8)—may affect the delivery efficiency. Surprisingly, we found that Cre protein can be simply and efficiently delivered into *Arabidopsis thaliana* cells (with up to 83% efficiency), even in the presence of a cell wall, by immersing *Arabidopsis thaliana* cells in an optimized buffer and performing electroporation. Since Cre forms a tetrameric complex with DNA in the recombination reaction, at least 4 molecules should be delivered into given a cell to facilitate this recombination reaction. Although further studies are needed to clarify the mechanism by which cells take in protein by analyzing the state of the cell wall and cell membrane immediately after electroporation, our finding that proteins can be delivered into *Arabidopsis thaliana* cells without removing the cell wall could considerably advance the field of plant genetics.

In conclusion, Cre recombinase, which is one of the most widely used genome engineering enzymes, was delivered into *Arabidopsis thaliana* cells through the cell wall with a high degree of efficiency. As Cre recombinase can induce site-specific recombination, this technology will enable the conditional knockout of lethal genes and the removal of unnecessary selection markers that adversely affect cell functions in cultured *Arabidopsis thaliana* cells. Research using cultured cells enables us to analyze gene functions more quickly and robustly with lesser variation at the level of the individual experiments. This simple, economical, and effective technology will contribute to the biological analysis of genes and cellular functions in cultured *Arabidopsis thaliana* cells, which has proven difficult to analyze thus far.

## Supplementary information

SUPPLEMENTARY INFO
